# Clinical Implications of Bacteremia Caused by Non-*baumannii Acinetobacter* Compared with Those of *Acinetobacter baumannii* Bacteremia

**DOI:** 10.3390/biomedicines13092304

**Published:** 2025-09-20

**Authors:** Jin Woong Suh, Ji Young Hong, Keun Ju Kim, Duck Jin Hong, Sun Bean Kim

**Affiliations:** 1 Department of Infectious Diseases, Kyung Hee University Hospital at Gangdong, Kyung Hee University College of Medicine, Seoul 05278, Republic of Korea; 2 Division of Pulmonary, Allergy and Critical Care Medicine, Department of Internal Medicine, Chuncheon Sacred Heart Hospital, Hallym University Medical Center, Chuncheon-si 24252, Republic of Korea; 3 Department of Laboratory Medicine, Korea University College of Medicine, Seoul 02841, Republic of Korea; 4 Department of Laboratory Medicine, Sheikh Khalifa Specialty Hospital, Ras Al Khaimah 6365, United Arab Emirates; 5 PureLab, Ras Al Khaimah 6365, United Arab Emirates; 6 International Vaccine Institute, SNU Research Park, Seoul 08826, Republic of Korea

**Keywords:** non-*baumannii Acinetobacter*, *A. baumannii*, bacteremia, association factor, 28-day mortality

## Abstract

**Objectives:** This study aimed to compare clinical characteristics, antimicrobial susceptibility, and 28-day mortality between patients with *Acinetobacter baumannii* bacteremia (ABB) and non-*baumannii Acinetobacter* bacteremia (NBAB) after rapid matrix-assisted laser desorption ionization time-of-flight (MALDI-TOF) mass spectrometry (MS) species identification. **Methods:** We retrospectively reviewed the clinical data of adult ABB and NBAB patients over >7 years. Multivariate logistic regression was used to identify the risk factors for 28-day mortality. **Results:** Of 273 episodes of *Acinetobacter* species bacteremia, 224 (82.1%) were ABB and 49 (17.9%) were NBAB. NBA isolates were predominantly *A. nosocomialis* (49%), with smaller proportions of *A. bereziniae*, *A. junii*, *A. ursingii*, and others. The primary sites of infection in NBAB cases were the intra-abdomen, urinary tract, intravascular catheters, and lungs. While only 4.0% of *A. baumannii* isolates were susceptible to carbapenem, 87.8% of non-*baumannii Acinetobacter* isolates were susceptible. Multivariate analysis revealed that low carbapenem resistance was independently associated with NBAB. Additionally, a higher Pitt bacteremia score, septic shock, continuous renal replacement therapy, inappropriate empirical antibiotic therapy, and thrombocytopenia were independent risk factors for the 28-day mortality in patients with ABB. **Conclusions:** Although less common than ABB, NBAB cases are increasing and exhibit lower carbapenem resistance. Rapid MALDI-TOF MS identification enables timely and appropriate antibiotic treatment. The key factors driving the 28-day mortality include illness severity, septic shock, renal replacement therapy, inappropriate antibiotics, and thrombocytopenia, highlighting the need for early risk assessments and tailored management. Ongoing surveillance and species-specific strategies are essential for combating resistant *Acinetobacter* infections.

## 1. Introduction

*Acinetobacter* species are generally regarded as part of the normal flora of human skin, mucous membranes of the pharynx, and human respiratory secretions. However, they can cause a wide range of local and systemic infections, including wound infections, pneumonia, and septicemia [1]. Risk factors for *Acinetobacter* infections include prolonged intensive care unit (ICU) stay, use of invasive medical devices, prior antimicrobial exposure, and underlying comorbidities, such as diabetes, malignancy, and chronic organ dysfunction [2,3]. The incidence of *A. baumannii* infections is overestimated in clinical laboratories because non-*baumannii Acinetobacter* species are frequently mistaken for *A*. *baumannii* owing to their strong phenotypic similarities [4,5].

In recent decades, *A. baumannii* has emerged as a clinically significant healthcare-associated pathogen, known for its multidrug resistance [5,6]. Compared to non-baumannii *Acinetobacter* species, *A. baumannii* is characterized by higher rates of carbapenem resistance and multidrug resistance and is more frequently associated with severe infections, such as pneumonia and septic shock. Contrarily, non-*baumannii* species tend to cause infections at different anatomical sites, exhibit relatively greater antimicrobial susceptibility, and are associated with lower mortality rates [7,8].

Although previous studies have suggested that non-*baumannii Acinetobacter* species rarely cause severe sepsis or septic shock [9], recent reports have documented serious infections due to multidrug-resistant *Acinetobacter pittii* and *Acinetobacter nosocomialis* in medical settings worldwide [10,11,12]. Moreover, *Acinetobacter calcoaceticus*, an environmental organism, has been isolated from patients with pneumonia and bacteremia [13], and nosocomial infections involving *Acinetobacter iwoffii*, *Acinetobacter junii*, and *Acinetobacter johnsonii* have also been reported [12].

The identification of *Acinetobacter* species using conventional biochemical methods remains challenging because of their limited discriminatory power [14,15]. The introduction of matrix-assisted laser desorption ionization time-of-flight (MALDI-TOF) mass spectrometry (MS) in clinical microbiology laboratories has significantly improved the analysis time and accuracy of species-level identification within the genus *Acinetobacter* [16,17,18]. Despite these advancements, few studies have systematically examined the clinical characteristics and significance of non-*baumannii Acinetobacter* infections identified by MALDI-TOF MS.

Therefore, this study aimed to assess and compare the clinical significance of non-*baumannii Acinetobacter* bacteremia (NBAB) and *Acinetobacter baumannii* bacteremia (ABB) by analyzing the differences in clinical features, antimicrobial resistance, and patient outcomes following the introduction of MALDI-TOF MS at a university-affiliated hospital in Seoul, South Korea, and to identify the risk factors for 28-day mortality among patients with *Acinetobacter* species bacteremia.

## 2. Materials and Methods

We compared the clinical features and antibiotic susceptibility of ABB and NBAB, and analyzed the risk factors for 28-day mortality among patients with *Acinetobacter* species bacteremia.

### 2.1. Study Population

A retrospective analysis was performed at a 1048-bed university-affiliated hospital in Seoul, South Korea, from December 2016 (time of MALDI-TOF MS implementation) to December 2023. Adult patients (≥18 years old) diagnosed with bacteremia caused by either *Acinetobacter baumannii* or non-*baumannii Acinetobacter* species that occurred ≥48 h after hospital admission, indicating that the infection was acquired within the healthcare facility, were included in this study. Patients’ demographic, microbiological, and clinical information were extracted from electronic medical records. These included age; sex; underlying diseases; predisposing conditions, such as mechanical ventilation, extracorporeal membrane oxygenation (ECMO), continuous renal replacement therapy (CRRT), ICU stay, history of antibiotic exposure and steroid use; infection source; appropriateness of empirical antibiotics; antimicrobial susceptibility of *Acinetobacter* species; laboratory data at the onset of bacteremia; Charlson comorbidity index [19]; Pitt bacteremia score [20]; and all-cause in-hospital mortality. Approval was obtained from the Institutional Review Board of Korea University Anam Hospital (approval number: 2023AN0257) before the inception of the study. Due to the retrospective nature of the study, the requirement for informed consent was waived.

### 2.2. Endpoints

The primary endpoint was the comparison of clinical features between patients with ABB and those with NBAB. The secondary endpoint was to evaluate the clinical significance, including risk factors for 28-day mortality in patients with *Acinetobacter* bacteremia, during the study period.

Blood culture, bacterial identification, and antimicrobial susceptibility testing.

Blood samples from patients clinically diagnosed with ABB/NBAB infections were inoculated into two pairs of blood culture bottles (Bactec Plus Aerobic and Bactec F Lytic Anaerobic; Becton Dickinson, Le Pont de Claix, France) and incubated in a BD Bactec 9240 blood culture system (BD Biosciences, Sparks, MD, USA) for at least 5 days. Positive cultures were subjected to direct Gram staining and subcultured on standard solid media for subsequent analyses. Bacterial colonies were initially identified using conventional biochemical methods (VITEK II bioMérieux, Marcy L’Etoile, France) [21]. Isolates initially identified as *Acinetobacter baumannii* did not require further confirmation. Non-*baumannii Acinetobacter* isolates were verified by MALDI-TOF MS (Bruker Daltonics GmbH, Billerica, MA, USA) using the Bruker MALDI Biotyper Compass Library version 4.0, following the manufacturer’s instructions [22]. Final confirmation of the species identification was performed using *rpoB* gene sequencing, which was analyzed using the Basic Local Alignment Search Tool (https://blast.ncbi.nlm.nih.gov/Blast.cgi, accessed on 2 June 2025) [23]. Accurate differentiation between AB and NBA species has been achieved using MALDI-TOF MS, which provides rapid and reliable species-level identification, and often outperforms conventional biochemical methods in terms of speed and accuracy [15,24].

The antibiotic susceptibility of *Acinetobacter* from blood cultures was determined using a MicroScan WalkAway-96 Plus system (Beckman Coulter, Inc., Brea, CA, USA). The susceptibility test results were interpreted per the Clinical Laboratory Standards Institute guidelines [23].

### 2.3. Definitions

Bacteremia was defined as positive cultures from two or more blood specimens drawn on separate occasions within 48 h, accompanied by at least one of the following clinical signs or symptoms at the time of blood sample collection: temperature > 38 °C, chills, or hypotension [25]. Patients with *Acinetobacter* bacteremia accompanied by clinical signs of infection consistent with sepsis, defined according to the Sepsis-3 criteria, were included. Septic shock was defined as persistent hypotension requiring vasopressors after fluid resuscitation [26]. We excluded patients with polymicrobial infections or infections arising from multiple anatomical sites to allow a clearer interpretation of the infection focus. Appropriate empirical antibiotic therapy was defined as the administration of at least one antimicrobial agent with in vitro susceptibility within 24 h of obtaining positive blood culture results and before susceptibility data became available [27]. Inappropriate empirical antibiotic therapy was defined as the administration of antibiotics lacking in vitro activity against the pathogen within 24 h of a positive culture. These include delayed initiation, suboptimal dosing, and use of agents with limited efficacy for bloodstream infections [28]. Thrombocytopenia was defined as a platelet count of <150,000/μL [29].

### 2.4. Statistical Analysis

SPSS software version 20 was used to conduct statistical analyses (IBM corp., Armonk, NY, USA) [30]. Student’s *t*-test was used to compare continuous variables, which were displayed as means ± standard deviations if normally distributed. The Mann–Whitney U test was used to evaluate non-normally distributed data, which were represented as medians with interquartile ranges (IQRs). Either Fisher’s exact test or the χ^2^ test was used to compare categorical variables. Logistic regression models with backward stepwise conditional selection were used for the multivariate analysis. To assess the correlations between variables, odds ratios (ORs) and their accompanying 95% confidence intervals (CIs) were computed. Statistical significance was defined as a two-sided *p*-value of <0.05.

## 3. Results

### 3.1. Study Population Characteristics

Table 1 shows the demographic information, clinical characteristics, and underlying conditions of all the patients with ABB and NBAB. Among the 273 patients, 167 (61.2%) were men, and the median age was 69 years (IQR, 60–78 years). The ABB and NBAB groups showed no significant differences in median age, sex, Charlson Comorbidity Index scores, or underlying comorbidities, except for cardiovascular disease. In univariate analysis, patients with ABB had a significantly higher prevalence of cardiovascular disease than those with NBAB. The primary site of infection in NBAB was the intra-abdomen (24.5%), urinary tract (18.4%), intravascular catheter (20.4%), lungs (16.3%), and skin and soft tissue (10.2%), whereas the primary site of infection in ABB was lungs (51.3%), intra-abdomen (11.6%), intravascular catheter (27.2%), skin and soft tissue (3.1%), and urinary tract (2.7%). Although 96.4% *A. baumannii* isolates were carbapenem-resistant, all non-*baumannii Acinetobacter* species isolates, except for eight, were carbapenem-susceptible (*p* < 0.001). Compared with patients with ABB, those with NBAB showed a significantly lower Pitt bacteremia score, rate of CRRT use, rate of antibiotic use within the preceding 30 days, occurrence of shock, rate of ICU stay at bacteremia onset, rate of inappropriate empirical antibiotic therapy, and 28-day mortality rate (Table 1).

### 3.2. Epidemiology of Acinetobacter Species Bacteremia

**Figure 1:**
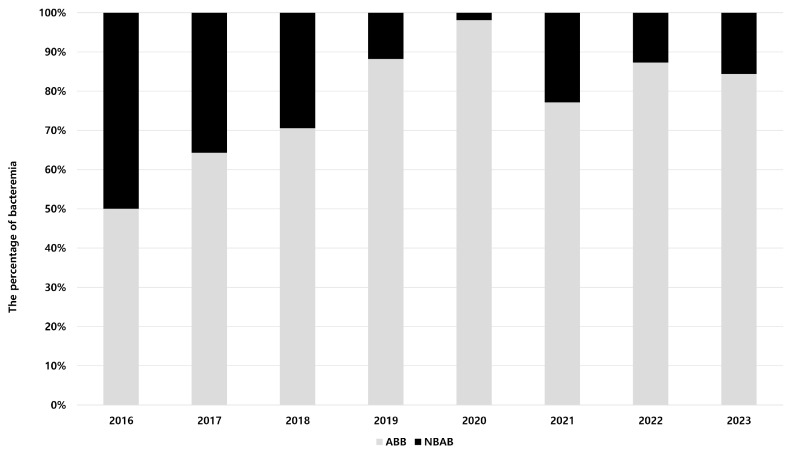
Annual distribution of *Acinetobacter* bacteremia cases by species during the study period.

A total of 379 episodes of *Acinetobacter* species bacteremia were identified; those involving catheter colonization (n = 67) and polymicrobial infection (n = 39) were excluded from the analysis. Of the remaining 273 episodes, 224 (82.1%) were ABB and 49 (17.9%) were NBAB. Figure 1 shows the changes in the proportions of ABB and NBAB cases over the study period. The number of ABB cases increased progressively from 50% in 2016 to 98.1% in 2020; the number of ABB cases increased progressively, accounting for most infections. From 2021 onwards, ABB will remain the dominant pathogen in patients with *Acinetobacter* bacteremia, consistently accounting for over 70% of cases annually. Regarding the distribution of non-*baumannii Acinetobacter* species during the study period, *A. nosocomialis* was the most common (49%, n = 24), followed by *A. bereziniae* (10.2%, n = 5), *A. ursingii* (8.2%, n = 4), and *A. junii* (8.2%, n = 4) (Table S1).

### 3.3. Antimicrobial Susceptibilities

The antimicrobial susceptibilities of *Acinetobacter species* clinical isolates are shown in Table 2. *A. baumannii* isolates were susceptible to minocycline (94.6%), tigecycline (71.2%), gentamicin (13.5%), trimethoprim-sulfamethoxazole (10.0%), ampicillin-sulbactam (5.4%), meropenem (4.0%), ceftazidime (4.0%), piperacillin-tazobactam (4.0%), and cefepime (4.0%) and showed intermediate susceptibility to colistin (72.5%). In contrast, non-*baumannii Acinetobacter* isolates were susceptible to tigecycline (100%), minocycline (97.8%), meropenem (87.8%), trimethoprim-sulfamethoxazole (87.5%), ampicillin-sulbactam (83.7%), piperacillin-tazobactam (81.6%), cefotaxime (69.4%), ceftazidime (65.3%), and cefepime (65.3%) and showed intermediate susceptibility to colistin (100%).

The eight carbapenem-resistant NBAB isolates patients exhibited significantly worse clinical outcomes and distinct characteristics than those with carbapenem-susceptible NBAB isolates. Septic shock was markedly more frequent in the carbapenem-resistant (CR)-NBAB group (62.5% vs. 4.9%, *p* < 0.001), as was ventilator use (37.5% vs. 9.8%, *p* = 0.040) and ventilator-associated pneumonia (12.5% vs. 0%, *p* = 0.022). The CR-NBAB group also had a substantially higher 28-day mortality rate (75.0% vs. 17.1%; *p* = 0.003). Moreover, inappropriate empirical antibiotic therapy was significantly more common among patients with CR-NBAB isolates (87.5% vs. 4.9%, *p* < 0.001), alongside a higher frequency of prior antibiotic use within 30 days (62.5% vs. 17.1%, *p* = 0.006). These findings highlight the severe clinical impacts and treatment challenges associated with carbapenem-resistant NBAB.

### 3.4. Factors Associated with NBAB

In multivariate analyses, carbapenem resistance was independently associated with a significantly lower incidence of NBAB (OR 0.007, 95% CI, 0.003–0.020; *p* < 0.001) (Table 3).

In the multivariate logistic regression model, a backward selection approach was adopted with variables including cardiovascular diseases, septic shock, ventilator, Pitt bacteremia score, CRRT, ICU stay at onset of bacteremia, inappropriateness of empirical antibiotics, prior antibiotic use in 30 days, carbapenem-resistant *Acinetobacter* spp., primary infection origin (urinary tract infection and pneumonia), WBC count, and albumin level (*p* < 0.05, univariate analysis).

### 3.5. Risk Factors for the 28-Day Mortality in Patients with Acinetobacter Species Bacteremia

The risk factors for 28-day mortality among patients with *Acinetobacter* bacteremia were analyzed during the study period (Table S2). The overall 28-day mortality rate from *Acinetobacter* species bacteremia was 50.5%. A significantly higher 28-day mortality rate was observed in patients with ABB than in those with NBAB (55.8% vs. 26.5%, *p* < 0.001). In multivariate analyses, the presence of *A. baumannii* or non-*baumannii Acinetobacter* was not identified as a significant factor associated with 28-day mortality in patients with *Acinetobacter* bacteremia. However, several independent risk factors for 28-day mortality with *A. baumannii* bacteremia were identified, including a higher Pitt bacteremia score (OR 1.148, 95% CI 1.031–1.278, *p* = 0.012), septic shock (OR 4.179, 95% CI 1.917–9.106, *p* < 0.001), CRRT (OR 2.525, 95% CI 1.148–5.555, *p* = 0.021), inappropriate empirical antibiotic therapy (OR 2.470, 95% CI 1.262–4.834, *p* = 0.008), and thrombocytopenia (OR 2.882, 95% CI 1.405–5.913, *p* = 0.004) (Table 4).

In the multivariate logistic regression model, a backward selection approach was adopted with variables including age, NBAB, Pitt bacteremia score, septic shock, ventilator, CRRT, Chronic liver disease, prior antibiotic use within 30 days, inappropriateness of empirical antibiotics, carbapenem-resistant *Acinetobacter* spp., primary infection origin (pneumonia, urinary tract infection, skin soft tissue infection), CRP, albumin, and thrombocytopenia (<150,000/μL) (*p* < 0.05, univariate analysis).

## 4. Discussion

Epidemiological trends emphasize the persistent predominance of ABB in hospital settings, despite the increasing recognition and isolation of NBA species. Annual data suggest that while interventions may have impacted the overall prevalence, AB remains the major cause of hospital-acquired *Acinetobacter* bacteremia [31,32]. Clinically, this reinforces the need for ongoing surveillance and species-level identification because the proportion of NBAB, although small, is not negligible and may have distinct susceptibility and outcome profiles. Furthermore, the annual increase in ABB cases highlights the ongoing challenge in managing multidrug-resistant infections and the need for targeted infection control strategies.

More than 50 species belonging to the genus *Acinetobacter* are regarded as non-pathogenic environmental organisms and are found in hospital sewage, soil samples around animal farms, and polluted rivers [33,34]. However, *Acinetobacter* species infections increased in frequency during the 1960s and the 1970s, coinciding with an increase in ICU admissions [35,36]. Initially, these species were considered commensal opportunists or low-virulence pathogens of limited clinical concern [34]. However, in the decades that followed, the prevalence of infections increased due to the widespread use of mechanical ventilation, central venous and urinary catheterization, and antimicrobial therapy [37]. In the present study, the incidence of pulmonary infections in patients with NBAB was significantly lower than that in patients with ABB. *A. baumannii* is well known for its propensity to colonize and infect the respiratory tract, especially in critically ill patients requiring mechanical ventilation in the ICUs, facilitating its role as a leading cause of ventilator-associated pneumonia [38,39]. In contrast, non-*baumannii* species, such as *A. nosocomialis* and *A. bereziniae*, are more frequently associated with infections at other anatomical sites, including intra-abdominal, urinary tract, and catheter-related infections, as reflected in the clinical data [40]. These observations are consistent with those of previous studies reporting species-specific infection patterns within the *Acinetobacter* genus [12,41,42].

MALDI-TOF MS is increasingly used in clinical microbiology laboratories to identify a wide range of microorganisms, including bacteria, yeast, and mycobacteria [16]. With the introduction of genospecies identification tools, such as MALDI-TOF MS, non-*baumannii Acinetobacter* species have been increasingly recognized as causes of bacteremia [43]. Compared to *A*. *baumannii*, non-*baumannii Acinetobacter* has a significantly different clinical relevance [15]. Although non-*baumannii* species tend to be less prevalent than *A. baumannii*, the distribution of *Acinetobacter* species varies by geographic region [9,10,11,12,44]. Several studies have reported the distribution of *Acinetobacter* spp. in clinical settings. A 3-year surveillance program found that *A. baumannii* (84.5%) was the predominant nosocomial species, whereas *A. pittii* accounted for only 5.6% of isolates [45]. Similar distributions were reported in surveillance studies conducted in Europe, Asia, and North America [33,44]. Consistent with these reports, we found *that A. baumannii* was the most commonly isolated, followed by *A. nosocomialis*, *A. bereziniae*, *A. ursingii*, and *A. junii*. However, regional variations in non-*baumannii Acinetobacter* bacteremia exist. *A. nosocomialis* and *A. pittii* represented a significant proportion of isolates from the United States (21% and 8%, respectively) [41]. Notably, in Norway, *A. nosocomialis* and *A. pittii* were found to cause bacteremia seven times more frequently than *A. baumannii*, highlighting the considerable geographic differences in species distribution [12].

In the present study, carbapenem resistance was more common in ABB than in NBAB. The rate of carbapenem resistance in the ABB group was higher than the national average estimate for South Korea (86%), but that of the NBAB group was lower than the national rate among NBAB isolates (32%) [46]. According to Korean surveillance data from 2020 to 2022, the proportion of carbapenem-resistant NBA isolates will increase from 8.2% in 2020 to 32% in 2022 [46]. Similarly, our study showed that the rate of carbapenem resistance among non-*baumannii Acinetobacter* isolates will increase from 0% in 2020 to 8.2% in 2022. This trend suggests a potential increase in carbapenem-resistant non-*baumannii Acinetobacter* over time, although the resistance rate remained lower than that of *A. baumannii* isolates. The increase in carbapenem resistance among AB strains is primarily attributed to challenges in hospital infection control, extensive use and misuse of antibiotics [47], and the horizontal transfer of resistance genes via mobile genetic elements, such as plasmids and transposons [48]. Environmental *Acinetobacter* strains, including *A. baumannii*, carry carbapenemase genes at rates ranging from approximately 8 to 40%, which is significantly lower than the prevalence of 75–90% observed in clinical *A. baumannii* isolates [49,50]. ESBL genes, such as blaCTX-M, blaTEM, blaSHV, and blaOXA-1, were detected in 2–45% of environmental *Acinetobacter* isolates [51,52]. Despite their lower prevalence compared to clinical strains, these environmental populations serve as important reservoirs for antimicrobial resistance genes with the potential for horizontal transfer and contribution to resistance dissemination [53]. Collectively, these factors drive the selection and proliferation of resistant clones.

Several studies have reported high colistin resistance in *A. nosocomialis*, *A. seifertii*, *A. bereziniae*, *A. calcoaceticus*, *A. junii*, and *A. baumannii* isolates [54,55]. The 2022 Korean national surveillance data indicate that 4.1% of *A. baumannii* and 12% of non-*baumannii Acinetobacter* isolates are resistant to colistin [46]. However, in our study, no non-*baumannii Acinetobacter* isolates were resistant to colistin, compared to 27.5% of *A. baumannii* isolates. This discrepancy may be attributed to institutional variations in circulating *Acinetobacter* strains with distinct genomic resistance profiles [56] and temporal shifts in resistance patterns linked to antibiotic stewardship policies and infection control practices [57]. These findings underscore the importance of continuous surveillance and strain-specific analysis for managing *Acinetobacter* bacteremia in clinical settings.

However, the characteristics of the NBAB have not been thoroughly investigated. A comparative study of bacteremia caused by *A. pittii* and *A. nosocomialis* found that patients with NBAB were more frequently exposed to chemotherapy and invasive procedures [42]. A previous study showed that ICU admission, mechanical ventilation, pneumonia, chronic liver disease, multidrug resistance, and carbapenem resistance are substantially more common in ABB, whereas NBAB exhibits a lower rate of carbapenem resistance than ABB [58]. Similarly, our study revealed that carbapenem resistance was independently associated with a lower risk of NBAB in the multivariate analysis. However, one study reported that NBA species can acquire carbapenem resistance genes via plasmids [59]. Therefore, comprehensive surveillance and rapid identification of antimicrobial resistance in both ABB and NBAB strains are essential to guide effective therapeutic interventions.

MALDI-TOF MS provides rapid and highly accurate identification of *Acinetobacter* species, outperforming conventional biochemical methods such as VITEK 2 and MicroScan WalkAway, which often struggle to differentiate closely related species owing to similar phenotypic profiles. Studies have shown that MALDI-TOF MS achieves an identification accuracy of above 90%, enabling timely and precise species-level diagnosis, which is critical for appropriate antimicrobial therapy and infection control [15,16,22,24]. Therefore, it facilitates timely clinical decision-making regarding strain-specific antimicrobial therapies. AB exhibited high levels of carbapenem resistance, whereas NBA demonstrated relatively higher antimicrobial susceptibility. This disparity resulted in significantly more inappropriate empirical antibiotic use in ABB patients compared to NBAB patients when using conventional biochemical methods. Utilizing MALDI-TOF MS to rapidly differentiate these species enables earlier and more appropriate empirical antibiotic selection, which can reduce unnecessary antibiotic exposure, improve patient outcomes, and curb the development and spread of antimicrobial resistance. Therefore, the integration of MALDI-TOF MS into routine clinical workflows supports strain-specific management strategies that optimize antimicrobial stewardship and patient care.

A significantly higher 28-day mortality rate was observed in patients who underwent ABB than in those who underwent NBAB. However, in logistic regression analysis, ABB was not identified as a significant predictor of 28-day mortality. Instead, the Pitt bacteremia score, septic shock, CRRT, inappropriate empirical antibiotic therapy, and thrombocytopenia were significantly associated with the 28-day mortality, regardless of the *Acinetobacter* species. Our findings align with those of previous studies that identified similar risk factors for mortality in *Acinetobacter* bacteremia. Previous reports have shown that shock, hemodialysis for acute kidney injury, absence of appropriate antibiotic therapy, and thrombocytopenia are associated with increased mortality in *Acinetobacter* complex bacteremia [60,61,62]. Similarly, Huang et al. identified a high Pitt bacteremia score as an independent prognostic indicator of mortality in patients with ABB [63]. Collectively, these studies highlight the critical role of illness severity at the onset of bacteremia in determining mortality outcomes in *Acinetobacter* infections.

Although NBAB infections are generally associated with better clinical outcomes and lower antimicrobial resistance than ABB infections [10], they may still progress to life-threatening complications such as septic shock and multi-organ failure, both of which are strongly associated with mortality [34,64]. Moreover, emerging antibiotic resistance in NBAB has been increasingly reported, particularly in the carbapenem-resistant strains of *A. nosocomialis* and *A. pittii* [65]. Regardless of the species, early recognition and management of severity indicators are essential for improving outcomes in *Acinetobacter* bacteremia. Therefore, the accurate and rapid species identification of *Acinetobacter* using MALDI-TOF MS enables clinicians to promptly tailor antimicrobial therapy according to species-specific resistance profiles. This approach facilitates earlier treatment optimization, reduces inappropriate antibiotic use, and ultimately improves clinical outcomes, emphasizing the importance of integrating species-level diagnostics and strain-specific management in routine clinical practice.

This study had several limitations. First, being a single-center retrospective study may limit the generalizability of our findings and introduce selection bias; to mitigate this, we included all consecutive cases over seven years. Second, the retrospective design could have led to incomplete or inaccurate data, potentially affecting the reliability of the results; however, we minimized this by using standardized data extraction and cross-checking records. Third, the small number of NBAB patients reduced the statistical power of the subgroup analyses, which remains a major drawback. Future multicenter studies with larger cohorts are required to validate our findings. Fourth, the unavailability of cefiderocol and aztreonam/avibactam in South Korea during the study period precluded the assessment of antimicrobial susceptibility to recently introduced last-resort agents. Fifth, this study is limited by the lack of detailed data on antibiotic regimens for patients with *Acinetobacter* bacteremia, which prevents the analysis of treatment effects on outcomes. Future research with comprehensive treatment information is needed to assess the antimicrobial efficacy.

## 5. Conclusions

This study highlights that while NBAB remains less common than ABB, its incidence is increasing in healthcare settings, and it is characterized by lower rates of carbapenem resistance. The use of MALDI-TOF MS for rapid and accurate species identification facilitates timely differentiation between ABB and NBAB, enabling appropriate empirical antibiotic selection and supporting antimicrobial stewardship. The key factors independently associated with increased 28-day mortality included higher Pitt bacteremia scores, septic shock, continuous renal replacement therapy, inappropriate empirical antibiotic therapy, and thrombocytopenia, underscoring the importance of early recognition and focused management of high-risk patients, regardless of species. These findings emphasize the need for ongoing surveillance and strain-specific treatment strategies to combat the threat of carbapenem-resistant *Acinetobacter* infections. Further large-scale multicenter studies are needed to confirm and extend these results.

## Figures and Tables

**Table 1:** Baseline clinical features and laboratory characteristics of the 273 patients with *Acinetobacter* species bacteremia.

Variables	Total (*n* = 273)	ABB (*n* = 224)	NBAB (*n* = 49)	*p*-Value
Median age, years (IQR)	69 (60–78)	69 (60–79)	69 (61–76)	0.419
Male n (%)	167 (61.2)	139 (62.1)	28 (57.1)	0.523
Comorbidities				
Diabetes mellitus, n (%)	116 (42.5)	97 (43.3)	19 (38.8)	0.561
Hypertension, n (%)	142 (52.0)	119 (53.1)	23 (46.9)	0.432
Cardiovascular disease, n (%)	60 (22.0)	55 (24.6)	5 (10.2)	0.028
Cerebrovascular disease, n (%)	61 (22.3)	48 (21.4)	13 (26.5)	0.437
Chronic kidney disease, n (%)	52 (19.0)	47 (21.0)	5 (10.2)	0.082
Chronic pulmonary disease, n (%)	22 (8.1)	20 (8.9)	2 (4.1)	0.259
Chronic liver disease, n (%)	25 (9.2)	20 (8.9)	5 (10.2)	0.779
Malignancy, n (%)				
Solid organ	103 (37.7)	81 (36.2)	22 (44.9)	0.253
Hematology	38 (13.9)	30 (13.4)	8 (16.3)	0.591
Organ transplantation, n (%)	10 (3.7)	9 (4.0)	1 (2.0)	0.505
Charlson comorbidity index (IQR)	3 (2–5)	3 (1–4)	3 (2–6)	0.127
Clinical severity				
Pitt bacteremia score (IQR)	3 (0–8)	4 (2–8)	0 (0–1)	<0.001
Sepsis, n (%)	138 (50.5)	96 (42.9)	42 (30.4)	<0.001
Septic shock, n (%)	135 (49.5)	128 (57.1)	7 (14.3)	<0.001
Mechanical ventilator, n (%)	155 (56.8)	148 (66.1)	7 (14.3)	<0.001
ECMO, n (%)	12 (4.4)	12 (5.4)	0 (0)	0.098
CRRT, n (%)	70 (25.6)	66 (29.5)	4 (8.2)	0.002
Risk factors				
ICU stay at onset of bacteremia, n (%)	162 (59.3)	151 (67.4)	11 (22.4)	<0.001
Steroid use in 90 days, n (%)	45 (46.5)	38 (17.0)	7 (14.3)	0.647
Prior antibiotic use in 30 days, n (%)	183 (67.0)	171 (76.3)	12 (24.5)	<0.001
Inappropriate empirical antibiotic therapy, n (%)	153 (56.0)	146 (65.2)	7 (14.3)	<0.001
Carbapenem-resistance, n (%)	224 (82.1)	216 (96.4)	8 (16.3)	<0.001
Primary infectious origin				
Pneumonia	123 (45.1)	115 (51.3)	8 (16.3)	<0.001
Intra-abdominal infection, n (%)	38 (13.9)	26 (11.6)	12 (24.5)	0.018
Urinary tract infection, n (%)	15 (5.5)	6 (2.7)	9 (18.4)	<0.001
Catheter related infection, n (%)	71 (26.0)	61 (27.2)	10 (20.4)	0.324
Skin and soft tissue infection, n (%)	12 (4.4)	7 (3.1)	5 (10.2)	0.029
Without primary infection origin, n (%)	23 (8.4)	18 (8.0)	5 (10.2)	0.621
All-cause mortality	171 (62.6)	155 (69.2)	16 (32.7)	<0.001
7-day	102 (37.4)	91 (40.6)	11 (22.4)	0.017
14-day	120 (44.0)	107 (47.8)	13 (26.5)	0.007
28-day	138 (50.5)	125 (55.8)	13 (26.5)	<0.001
60-day	152 (55.7)	136 (60.7)	16 (32.7)	<0.001
Laboratory parameters				
WBC count (×10^3^/µL)	10.1 (6.2–16.8)	10.7 (6.2–17.6)	7.6 (6.4–11.6)	0.039
PLT count (×10^3^/µL)	108 (48–188)	103.5 (46.0–179.0)	145 (62–196.5)	0.332
CRP (nmol/L)	99.2 (46.0–161.6)	101.6 (51.4–162.4)	89.7 (27.7–161.6)	0.607
PCT (ng/mL)	1.26 (0.34–5.69)	1.34 (0.367–6.38)	0.60 (0.13–1.87)	0.607
Albumin (mg/dL)	2.7 (2.4–3.0)	2.7 (2.4–2.9)	3.1 (2.6–3.6)	<0.001

Abbreviations: ABB, *Acinetobacter baumannii* bacteremia; NBAB, non-*baumannii Acinetobacter* bacteremia; IQR, interquartile range; ECMO, extracorporeal membrane oxygenation; CRRT, continuous renal replacement therapy; ICU, intensive care unit; WBC, white blood cell; PLT, platelet; CRP, C-reactive protein; PCT, procalcitonin; spp., species. Data are expressed as medians (interquartile ranges) and numbers (percentages).

**Table 2:** Antibiotic susceptibility of 221 patients with *Acinetobacter* species of bacteremia.

Resistance, n (%)	Total (*n* = 273)	ABB (*n* = 224)	NBAB (*n* = 49)	*p*-Value
Ampicillin-sulbactam, (n = 273)	220 (80.6)	212 (94.6)	8 (16.3)	<0.001
Piperacillin-tazobactam, (n = 273)	224 (82.1)	215 (96.0)	9 (18.4)	<0.001
Cefotaxime, (n = 273)	234 (85.7)	219 (97.8)	15 (30.6)	<0.001
Ceftazidime, (n = 273)	232 (85.0)	215 (96.0)	17 (34.7)	<0.001
Cefepime, (n = 273)	232 (85.0)	215 (96.0)	17 (34.7)	<0.001
Aztreonam, (n = 191)	179 (93.7)	152 (99.3)	27 (71.1)	<0.001
Imipenem, (n = 265)	221 (83.4)	215 (96.4)	6 (14.3)	<0.001
Meropenem, (n = 273)	221 (81.0)	215 (96.0)	6 (12.2)	<0.001
Gentamicin, (n = 218)	166 (76.1)	147 (86.5)	19 (39.6)	<0.001
Ciprofloxacin, (n = 273)	233 (85.3)	215 (96.0)	18 (36.7)	<0.001
Minocycline, (n = 268)	13 (4.9)	12 (5.4)	1 (2.2)	0.353
Tetracycline, (n = 54)	28 (51.9)	27 (84.4)	1 (4.5)	<0.001
Tigecycline, (n = 220)	55 (25.0)	55 (28.8)	0 (0)	0.001
Trimethoprim-sulfamethoxazole, (n = 218)	159 (72.9)	153 (90.0)	6 (12.5)	<0.001
Colistin, (n = 133)	28 (21.1)	28 (27.5)	0 (0)	0.001
Tobramycin, (n = 41)	28 (68.3)	20 (83.3)	8 (47.1)	0.014
Amikacin, (n = 48)	24 (50.0)	18 (72.0)	6 (26.1)	0.001

Abbreviations: ABB, *Acinetobacter baumannii* bacteremia; NBAB, non-*baumannii Acinetobacter* bacteremia.

**Table 3:** Multivariate analysis of association factors for non-*baumannii Acinetobacter* bacteremia.

Variables	Odds Ratio	95% CI	*p*-Value
Carbapenem-resistance	0.007	0.003–0.020	<0.001

Abbreviations: CI, confidence interval.

**Table 4:** Risk factors for the 28-day mortality in patients with *Acinetobacter* species bacteremia.

Variables	Odds Ratio	95% CI	*p*-Value
Septic shock	4.179	1.917–9.106	<0.001
Pitt bacteremic score	1.148	1.031–1.278	0.012
CRRT	2.525	1.148–5.555	0.021
Inappropriate empirical antibiotic therapy	2.470	1.262–4.834	0.008
Thrombocytopenia	2.882	1.405–5.913	0.004
Age	1.024	0.998–1.050	0.068
Primary infection origin (urinary tract infection)	0.209	0.024–1.822	0.156
Primary infection origin (skin soft tissue infection)	0.180	0.018–1.802	0.144

Abbreviations: CRRT, continuous renal replacement therapy; CI, confidence interval.

## Data Availability

The datasets generated or analyzed in the current study are available from the corresponding author upon reasonable request.

## Supplementary Materials

The following supporting information can be downloaded at https://www.mdpi.com/article/10.3390/biomedicines13092304/s1, Table S1: Species of non-*baumannii Acinetobacter* bacteremia; Table S2: Comparison of the clinical features associated with 28-day mortality of the 273 patients with *Acinetobacter* species bacteremia.
