# Supplementary material for: Clinical Implications of Bacteremia Caused by Non-baumannii Acinetobacter Compared with Those of Acinetobacter baumannii Bacteremia
Source: Biomedicines. 2025 Sep 20;13(9):2304. doi: 10.3390/biomedicines13092304 (PMC12467366; doi:10.3390/biomedicines13092304)
Supplement: Supplementary file 1 [file biomedicines-13-02304-s001.zip › biomedicines-3846883-supplementary.pdf]

Table S1. Species of non-*baumannii Acinetobacter* bacteremia

Species	Number (%)
A. nosocomialis	24 (49)
A. ursingii	4 (8.2)
A. bereziniae	5 (10.2)
A. pittii	2 (4.1)
A. baylyl	2 (4.1)
A. lwoffii	2 (4.1)
A. radioresistens	2 (4.1)
A. haemolyticus	2 (4.1)
A. junii	4 (8.2)
A. seifertii	2 (4.1)
Total	49 (100)

Table S2. Comparison of the clinical features associated with 28-days mortality of the 273 patients with *Acinetobacter* species bacteremia

Variables	Total (*n* = 273)	Survivors (*n* = 135)	Deaths (*n* = 138)	*p*-value
Median age, years (IQR)	69 (60–78)	68 (58–74)	72 (61–81)	0.016
Male (%)	167 (61.2)	87 (64.4)	80 (58.0)	0.273
NBAB, n (%)	49 (17.9)	36 (26.7)	13 (9.4)	<0.001
**Comorbidities**				
Diabetes mellitus, n (%)	116 (42.5)	57 (42.2)	59 (42.8)	0.929
Hypertension, n (%)	142 (52.0)	72 (53.3)	70 (50.7)	0.666
Cardiovascular disease, n (%)	60 (22.0)	26 (19.3)	34 (24.6)	0.283
Cerebrovascular disease, n (%)	61 (22.3)	35 (25.9)	26 (18.8)	0.160
Chronic kidney disease, n (%)	52 (19.0)	22 (16.3)	30 (21.7)	0.252
Chronic pulmonary disease, n (%)	22 (8.1)	13 (9.6)	9 (6.5)	0.346
Chronic liver disease, n (%)	25 (9.2)	7 (5.2)	18 (13.0)	0.024
Malignancy, n (%)				
- Solid organ	103 (37.7)	46 (34.1)	57 (41.3)	0.218
- Hematology	38 (13.9)	19 (14.1)	19 (13.8)	0.942
Organ transplantation, n (%)	10 (3.7)	4 (3.0)	6 (4.3)	0.543
Charlson comorbidity index (IQR)	3 (2–5)	3 (1–4)	3 (2–5)	0.108
**Clinical severity**				
Pitt bacteremia score (IQR)	3 (0–8)	1 (0–4)	8 (3–8)	<0.001
Shock, n (%)	135 (49.5)	26 (19.3)	109 (79.0)	<0.001
Mechanical ventilator, n (%)	155 (56.8)	55 (40.7)	100 (72.5)	<0.001
ECMO, n (%)	12 (4.4)	4 (3.0)	8 (5.8)	0.253
CRRT, n (%)	70 (25.6)	16 (11.9)	54 (39.1)	<0.001
**Risk factors**				
ICU stays at onset of bacteremia, n (%)	162 (59.3)	76 (56.3)	86 (62.3)	0.311

Steroid use in 90 days, n (%)	45 (16.5)	20 (14.8)	25 (18.1)	0.462
Prior antibiotics use in 30 days, n (%)	183 (67.0)	78 (57.8)	105 (76.1)	0.001
Inappropriate empirical antibiotic therapy, n (%)	153 (56.0)	52 (38.5)	101 (73.2)	<0.001
Carbapenem-resistance, n (%)	224 (82.1)	96 (71.1)	128 (92.8)	<0.001
**Primary infectious origin**				
Pneumonia	123 (45.1)	49 (36.3)	74 (53.6)	0.004
Intra-abdominal infection, n (%)	38 (13.9)	19 (14.1)	19 (13.8)	0.942
Urinary tract infection, n (%)	15 (5.5)	14 (10.4)	1 (0.7)	<0.001
Catheter related infection, n (%)	71 (26.0)	32 (23.7)	39 (28.3)	0.391
Skin and soft tissue infection, n (%)	12 (4.4)	11 (8.1)	1 (0.7)	0.003
**Laboratory parameters**				
WBC count (x10^3/μL)	10.1 (62.2–16.8)	9.7 (6.7–14.7)	10.4 (5.8–18.4)	0.123
PLT count (x10^3/μL)	108 (48 – 188)	159 (9– 234)	61 (38.8–112)	<0.001
CRP (nmol/L)	99.2 (46.0–161.6)	84.8 (38.1–146.1)	116.1 (54.9–171.4)	0.002
PCT (ng/mL)	1.26 (0.34–5.69)	0.52 (0.20–1.89)	2.44 (0.79–9.58)	0.174
Albumin (mg/dL)	2.7 (2.4–3.0)	2.8 (2.5–3.2)	2.6 (2.3–2.9)	<0.001

Abbreviations: IQR, interquartile range; PCT, procalcitonin; CRP, C-reactive protein; PLT, platelet; WBC, white blood cell; ICU, intensive care unit; ECMO, extracorporeal membrane oxygenation; CRRT, continuous renal replacement therapy; NBAB, non-*baumannii Acinetobacter* bacteremia
